# Supplementary material for: Primary cilia control cellular patterning of Meibomian glands during morphogenesis but not lipid composition
Source: Commun Biol. 2023 Mar 17;6:282. doi: 10.1038/s42003-023-04632-5 (PMC10023665; doi:10.1038/s42003-023-04632-5)
Supplement: Supplementary file 2 — Supplementary Information [file 42003_2023_4632_MOESM2_ESM.pdf]

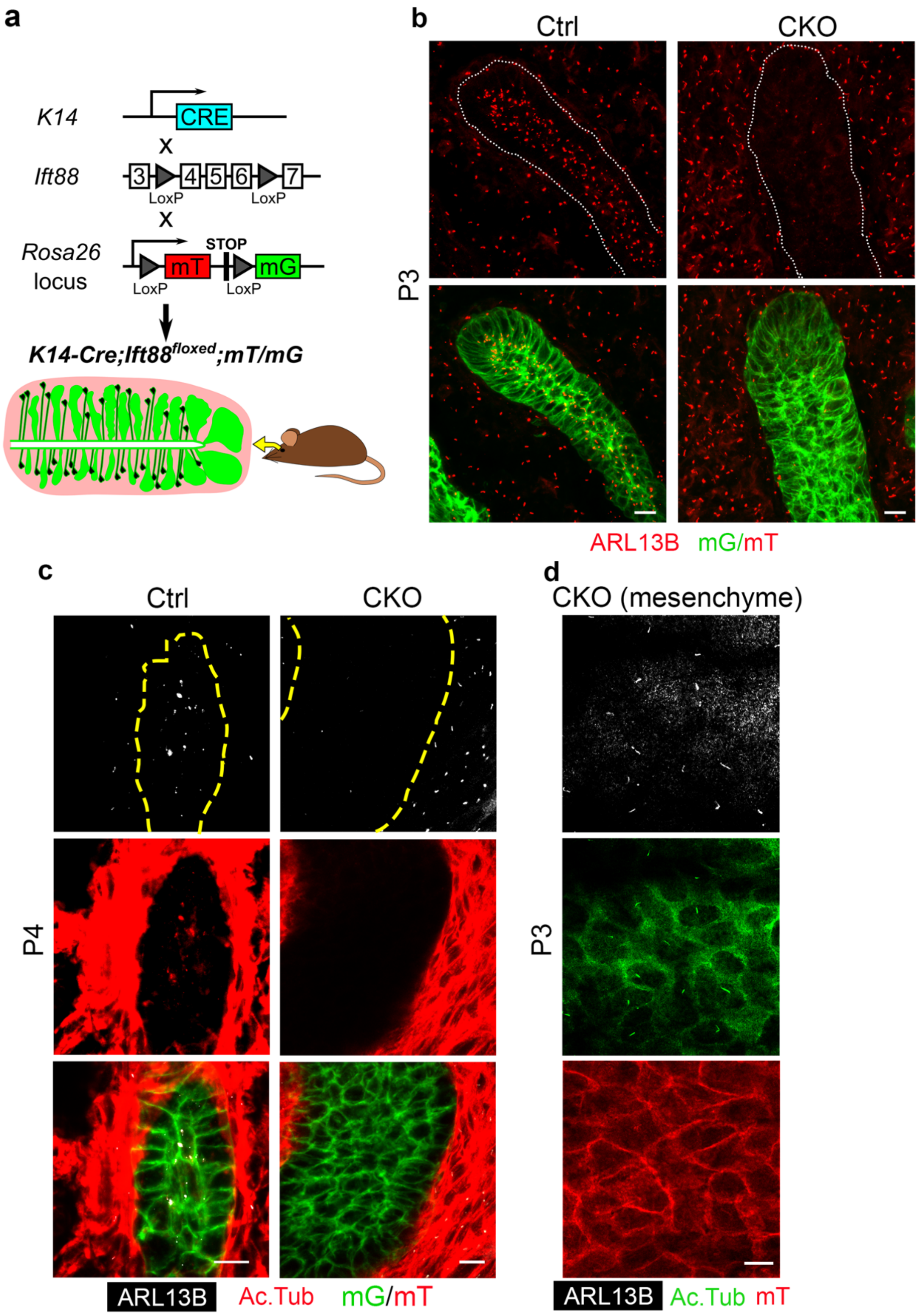

Supplement Figure 1

Supplement figure 1: Genetic deletion of *Ift88* in K14-expressing cells leads to primary cilium ablation in MGs. (**a**) Breeding strategy to generate conditional ciliary mutant and visualize Cre expression. (**b**) Representative MG sections of control and cKO mice at P3. MGs (surrounded by white dotted line) express the mG reporter. Primary cilia were stained with an anti-ARL13B antibody. Scale bar; 10 µm. (**c**) Representative MG sections of control and cKO mice at P3. MGs (surrounded by yellow dashed line) express the mG reporter. Primary cilia were stained with an anti-acetylated tubulin (Ac. Tuc) and an anti-ARL13B antibodies. Scale bar; 10 µm. (**d**) Representative section of the mesenchyme surrounding the MGs in cKO mice at P3. The mesenchyme expresses the mT reporter. Primary cilia were stained with an anti-acetylated tubulin (Ac. Tuc) and an anti-ARL13B antibodies. Scale bar; 10 µm.

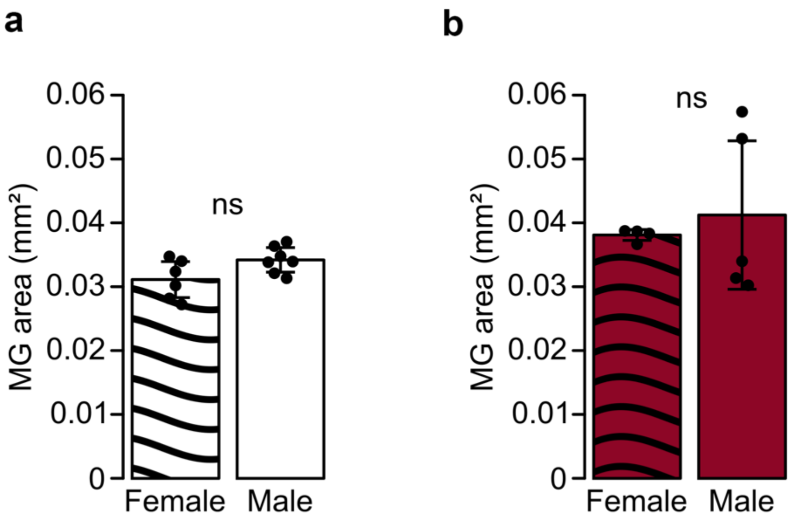

Supplement Figure 2

Supplement figure 2: MG size is not sex-dependent during MG development. MGs were stained with ORO in whole mount tarsal plates females in control (**a**) and cKO (**b**) mice at P8. Per mouse, MG area was determined by averaging the MG area of all individual MGs in the upper and lower eyelids. Data were presented as mean ± SD (n=6 females and 7 males for control mice; n=4 females and 5 males for cKO mice). Statistical significance was assessed using Mann Whitney test. ns, non-significant, $P \geq 0.05$.

a

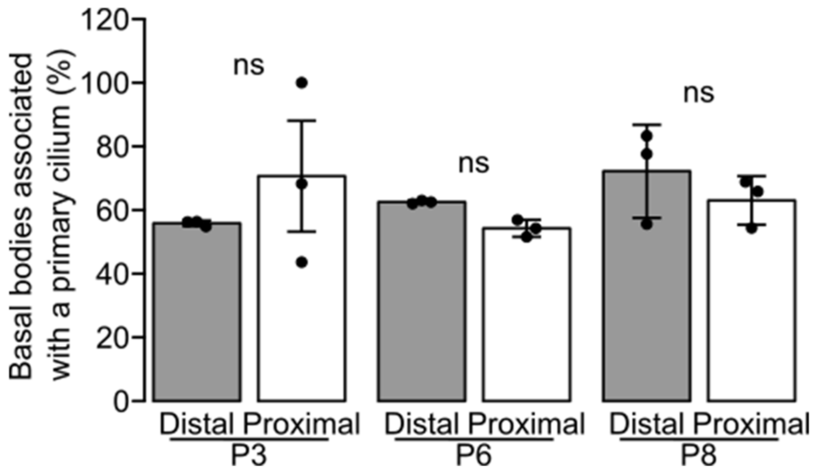

Supplement Figure 3

Supplement figure 3: Primary cilia are homogeneously distributed with MGs during early steps of MG development. The percentage of basal bodies associated with a primary cilium was quantified in the distal half and in the proximal half of MGs at P3, P6 and P8 (n=3 for each age). Data were presented as mean ± SD. Statistical significance was assessed using Wilcoxon signed rank test (distal vs proximal). ns, non-significant, $P \geq 0.05$.

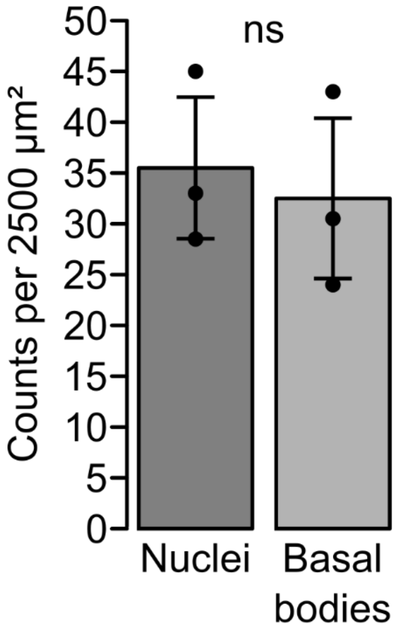

Supplement Figure 4

Supplement figure 4: The number of basal bodies is similar to the number of nuclei in MGs. Basal bodies and nuclei were counted in 50x50 µm areas on P25 MG sections. Two different areas including ductal and acini parts of the same gland were quantified and averaged (n=3 mice). Data were presented as mean ± SD. Statistical significance was assessed using Wilcoxon signed rank test (distal vs proximal). ns, non-significant, $P≥0.05$.
